# CRISPR/Cas9-Targeted Gene Editing of Allergenic Profilin-Encoding *Lyc e1* in Tomato Fruit

**DOI:** 10.3390/plants14243837

**Published:** 2025-12-16

**Authors:** Fanzhuang Yan, Jian Yao, Myungjin Lee, Ok-Jae Koo, Geung-Joo Lee

**Affiliations:** 1 Department of Smart Agriculture Systems, Chungnam National University, Daejeon 34134, Republic of Korea; yanfanzhuang@126.com (F.Y.); yaojiansdau@163.com (J.Y.); 2 Department of Horticulture, Chungnam National University, Daejeon 34134, Republic of Korea; leemj@cnu.ac.kr; 3 Department of Veterinary Medicine, Kyungpook National University, Daegu 41566, Republic of Korea; okjae.koo@gmail.com

**Keywords:** *Agrobacterium*-mediated transformation, Cas9-Free, CRISPR/Cas9, profilin, tomato allergy

## Abstract

Tomato (*Solanum lycopersicum* L.), one of the most widely consumed horticultural crops worldwide, is also a notable source of food allergens, with a higher prevalence of allergic reactions in pollen-sensitized patients. Profilin, a small actin-binding protein, is a major allergen in tomato fruits encoded by two homologous genes, *Lyc e1.01* and *Lyc e1.02*. Although various strategies have been proposed to reduce allergenicity in foods, no prior study has successfully achieved the complete elimination of profilin proteins in tomato using precise genome editing. In this study, we designed a single-guide RNA targeting a conserved region of both genes and employed the CRISPR/Cas9 system to generate loss-of-function mutations. We first evaluated single-guide RNA editing efficiency in tomato protoplasts and then performed *Agrobacterium*-mediated stable transformation, which yielded 13 transgenic T_0_ lines. Genotyping and Western blot analyses confirmed successful editing at both target loci and significantly decreased profilin accumulation in mutant tomato fruits. Notably, two homozygous Cas9-free lines were identified in the T1 generation, and one of these (line 23-15) showed significantly decreased profilin protein levels in the fruit. These findings demonstrate that the CRISPR/Cas9-mediated disruption of allergenic *Lyc e1* genes effectively removes profilin from tomato fruits. This strategy provides a promising framework for developing hypoallergenic tomato cultivars that may be extended to other related crop species.

## 1. Introduction

Tomato (*Solanum lycopersicum* L.) is an important fruit crop valued not only for its nutritional benefits but also for its versatility in fresh and processed forms, such as juice, sauce, and paste. Despite their widespread consumption, tomatoes are a known source of food allergens, particularly in individuals sensitized to birch pollen owing to cross-reactivity [1]. Reactions range from mild oral allergy syndrome to severe respiratory symptoms [2]. Thus, reducing the allergenic potential of tomatoes is an important goal for food safety and consumer health.

At least seven allergenic proteins have been identified in tomato, including profilin, β-fructofuranosidase, nonspecific lipid transfer proteins 1 and 2, Bet v 1-homologous PR-10 proteins, cyclophilin, and additional isoforms of nonspecific lipid transfer protein 1 [3,4,5,6,7,8]. Among these, profilin is a major allergen encoded by two closely related genes, *Lyc e1.01* (*Solyc08g066110.3.1*) and *Lyc e1.02* (*Solyc11g070130.2.1*). Previous studies using RNA interference to silence *Lyc e1* and *Lyc e3* achieved a partial reduction in profilin expression and alleviated allergic responses [5,9], suggesting the potential for genetic interventions that target these loci.

The CRISPR/Cas9 system offers a powerful and precise platform for targeted genome editing. Compared to earlier tools, such as zinc-finger nucleases and transcription activator-like effector nucleases, CRISPR/Cas9 is more efficient, cost-effective, and easier to design [10]. This system induces site-specific double-strand breaks, which are repaired by error-prone non-homologous end-joining or homology-directed repair, often resulting in indels that disrupt gene function [11,12].

CRISPR/Cas9 technology has been successfully applied to a wide range of crop species, including *Arabidopsis*, rice (*Oryza sativa*), maize (*Zea mays* L.), wheat (*Triticum aestivum*), soybean (*Glycine max*), and various horticultural plants, such as petunia (*Petunia* × *hybrida)*, *citrus*, and tomatoes [13,14,15,16,17,18]. Notably, the CRISPR/Cas9-mediated knockout of allergenic genes has demonstrated great success in several plant species, including peanuts (*Arachis hypogaea* L.), soybeans, and brown mustards. In peanuts, targeted disruption of the *2S albumin* gene led to a significant reduction in allergen levels compared to wild-type peanuts [19]. In soybeans, the allergenic genes *Gly m Bd 28K* and *Gly m Bd 30K* were successfully knocked out, and immunoblotting analysis confirmed that the edited plants no longer produced the corresponding allergenic proteins [20]. Similarly, in brown mustard (*Brassica juncea*), the major allergen *Bra j 1* was effectively eliminated, with edited seeds showing a substantial decrease in Bra j 1 protein levels, and a complete loss of expression in some lines [21]. These findings highlight the potential of CRISPR/Cas9 as a powerful tool for developing allergen-free tomato cultivars.

Protoplast-based assays are frequently used to validate CRISPR/Cas9 editing efficacy before generating stable transformants. In addition to confirming the functionality of CRISPR/Cas9 constructs, protoplast systems allow the rapid and efficient evaluation of single-guide RNA (sgRNA) editing efficiency, facilitating the selection of highly effective sgRNAs [22]. In some cases, protoplast editing has enabled the regeneration of DNA-free homozygous mutants [23,24,25].

Although profilin has been identified as a major allergen in tomato fruits [26], effective strategies to eliminate its expression through precise genome editing have not yet been established. Previous studies have primarily focused on characterizing tomato allergens or using RNA interference approaches, which often result in incomplete or transient suppression of allergenic proteins [9]. To date, no research has successfully achieved stable, heritable knockout of the profilin-encoding genes *Lyc e1.01* and *Lyc e1.02* in tomato using CRISPR/Cas9.

In this study, we assessed the editing efficiency of an sgRNA targeting a conserved region in both *Lyc e1.01* and *Lyc e1.02* genes using tomato protoplasts. Subsequently, we generated transgenic plants via *Agrobacterium*-mediated transformation, in which homozygous Cas9-free lines without profilin proteins were generated in post-generations. Our results provide a framework for developing hypoallergenic tomato cultivars and have broad applications for *Solanaceae* crop improvement.

## 2. Results

**Figure 1:**
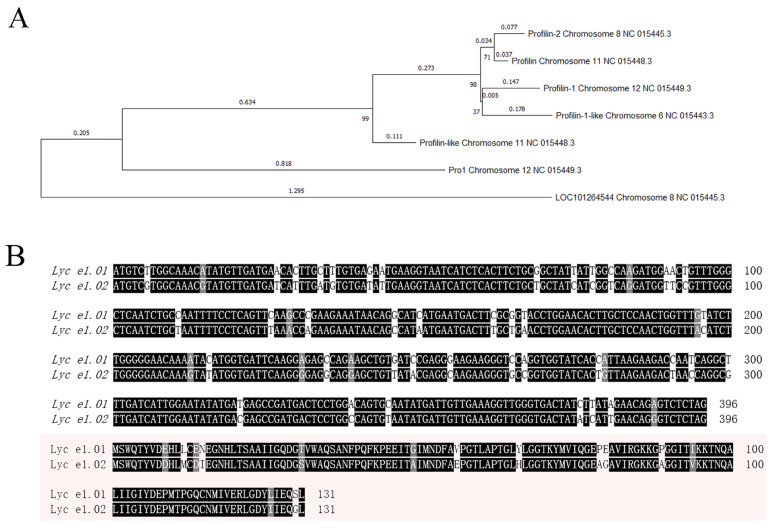
Phylogenetic relationship and sequence alignment of tomato profilin genes. (**A**) Phylogenetic tree of the seven tomato profilin family members constructed using the Neighbor-Joining method in MEGA11. Bootstrap values (1000 replicates) are shown. *Profilin-2* (*Lyc e1.01*) and *Profilin* (*Lyc e1.02*), highlighted in the tree, are known tomato allergens involved in fruit allergenicity. (**B**) Nucleotide and amino acid sequence alignment of *Lyc e1.01* and *Lyc e1.02*. The nucleotide and amino acid sequence identities between the two genes are 87.9% and 89.3%, respectively, highlighting their high degree of conservation. To achieve simultaneous knockout of both genes, the sgRNA was designed to target a conserved region. Black indicates identical residues, gray indicates conservative substitutions, and white indicates differences.

The tomato profilin protein family includes seven members, including profilin-2 and profilin, encoded by *Lyc e1.01* and *Lyc e1.02*, respectively, which are major allergens (Figure 1A). These genes shared high sequence identity of 87.9% at the nucleotide level and 89.3% at the amino acid level (Figure 1B). A conserved region common to both genes was identified using the CRISPRdirect tool, and a single sgRNA was designed to simultaneously target both loci.

The sgRNA target site for *Lyc e1* was identified at nucleotide positions 346–368 on the positive strand (Table 1). The selected target sequence was 20 bp long, followed by a PAM motif. The out-of-frame score, which reflects the likelihood of inducing a frameshift mutation upon cleavage, was 62.6. Off-target analysis revealed two potential off-target sites with zero mismatches, and one with one mismatch, indicating a moderate risk of off-target effects for this sgRNA design. Notably, the two perfect-match (zero-mismatch) sites corresponded to homologous regions within the target genes, suggesting that the sgRNA design simultaneously targeted both gene copies.

The editing efficiencies of CRISPR-induced indels were evaluated for *Lyc e1.01* and *Lyc e1.02* genes by introducing the prepared ribonucleoproteins into tomato protoplasts (Table 2). The sgRNA was synthesized and complexed with Cas9 protein to form ribonucleoproteins, which were delivered into tomato protoplasts using PEG-Ca^2+^-mediated transformation. Approximately 64% of tomato protoplasts transformed with the GFP plasmid showed green fluorescence (Figure S1), indicating that the RNP transformation was also successful. Deep sequencing revealed successful editing at both target sites, with efficiencies of 6.1% (536 mutated reads out of 8836 reads) for *Lyc e1.01* and 42.5% (10,019 mutated reads from 23,569 reads) for *Lyc e1.02*. These differences may reflect variations in chromatin accessibility or locus-specific repair dynamics. For *Lyc e1.01*, three types of indels (+G insertion, −A deletion, +A insertion) were detected at frequencies of 2.31%, 2.2%, and 0.68%, respectively. For *Lyc e1.02*, three different indel types (two +G insertions, −GAAA deletion) were observed in 10,019 mutated reads from 23,569 reads (total 42.5%), with mutation rates of 12.9%, 5.75%, and 1.93%, respectively. Again, these differences may reflect variations in chromatin accessibility or locus-specific repair dynamics.

**Figure 2:**
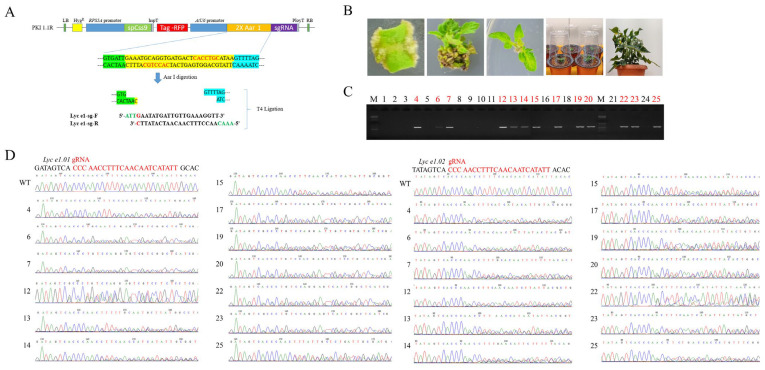
CRISPR/Cas9 vector construction and editing analysis in T_0_ tomato plants. (**A**) Cloning strategy of the sgRNA into the pKI1.1R binary vector using T4 DNA ligase. The plasmid was linearized using the AarI restriction enzyme. ATTG and AAAC sequences were added to the 5′ and 3′ ends of the target sequence, respectively, and the fragment was inserted via homologous recombination. Red letters indicate the AarI restriction enzyme recognition site; green and blue highlighted regions represent the sticky ends generated after upstream and downstream digestion, respectively. The sgRNA sequence is highlighted in yellow. (**B**) Workflow of *Agrobacterium*-mediated transformation and regeneration in tomato. The process includes explant preparation, *Agrobacterium* infection, co-cultivation, callus induction, shoot regeneration, rooting, and acclimatization of regenerated plants. (**C**) PCR confirmation of Cas9 integration in regenerated plants. Cas9-specific primers were used to amplify the transgene region. Among 25 regenerated lines, 13 showed a clear Cas9 amplicon, indicating successful transgene integration (lines 4, 6, 7, 12, 13, 14, 15, 17, 19, 20, 22, 23, and 25 in red font). These lines were considered transgenic-positive. M: molecular marker; 1–25: regenerated lines. (**D**) Sanger sequencing chromatograms of target loci in wild type and positive lines. Mixed sequencing signals (overlapping peaks) appeared 3–4 bp upstream of the PAM site, consistent with the occurrence of insertion/deletion mutations introduced by CRISPR/Cas9. No overlapping peaks were observed in wild-type controls, confirming the specificity of the detected edits.

A total of 25 T_0_ lines were regenerated via *Agrobacterium*-mediated transformation, with 13 confirmed as transgenic (52%) (Table 3, Figure 2C). Genotyping revealed that all 13 lines harbored mutations in the target loci (Table 4). However, most lines were chimeric, with only a few exhibiting biallelic or homozygous mutations: 15.4% for *Lyc e1.01* and 7.7% for *Lyc e1.02*.

Biallelic mutations were observed in line 4, line 23, and line 25; line 4 had deletions of 1 or 2 bp in *Lyc e1.01*, whereas line 25 carried deletions of 2 or 11 bp (Table 4). In contrast to the chimeric mutation types for all other T_0_ lines, T_0_ line 23, which showed no profilin production, exhibited two distinct chimeric deletions (−8 and −24 bp) in *Lyc e1.01* and one of −4 bp in *Lyc e1.02*. Self-pollination of T_0_ line 23 led to Cas9-free, homozygous mutants in the T_1_ generation.

Inheritance of mutated alleles in the next generation was also evaluated. T_0_ line 15 developed fruit, but its seeds were unable to germinate because the pollen was inviable. From 12 fertile T_0_ lines, 240 T_1_ plants were obtained, with 20 individual plants propagated per line to ensure a representative sampling of each transformation event. PCR analysis revealed 50 Cas9-free plants (Figure S2), representing a frequency lower than the 25% expected under Mendelian segregation. This deviation is likely due to the presence of multiple T-DNA copies in certain T_0_ lines, which can complicate segregation patterns and reduce the proportion of null segregants in the T_1_ generation. Among these, 23 plants were homozygous for mutations in *Lyc e1.01,* and five were homozygous for mutations in *Lyc e1.02*. Two lines, 23-9 and 23-15, were homozygously mutated in both *Lyc e1.01* and *Lyc e1.02* genes and were Cas9-free (Table S1).

**Figure 3:**
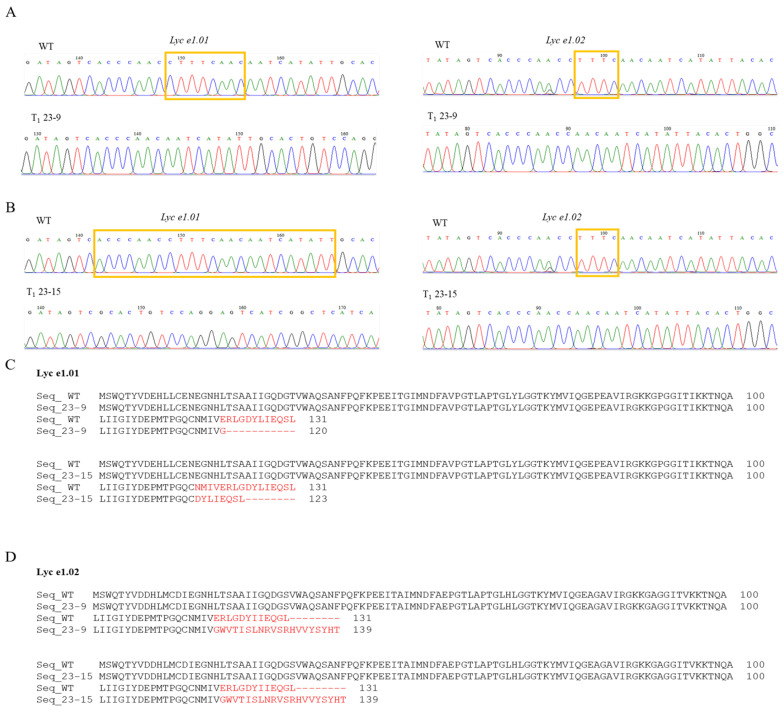
Mutation genotyping and predicted protein changes in Cas9-free homozygous T_1_ lines. (**A**) Sanger sequencing chromatograms and indel analysis of CRISPR-edited line 23-9. An 8 bp deletion with 100% mutation frequency was detected at the *Lyc e1.01* locus, and a 4 bp deletion with 99% efficiency was observed at the *Lyc e1.02* locus. The base deletion in the mutant is boxed in yellow. (**B**) Sanger sequencing chromatograms and indel analysis of line 23-15. The *Lyc e1.01* locus exhibited a homozygous 24 bp deletion (100% mutation frequency), and the *Lyc e1.02* locus showed a 4 bp deletion with a mutation efficiency of 99%. The base deletion in the mutant is boxed in yellow. (**C**) Deletion patterns in Lyc e1.01: an 8 bp deletion in line 23-9 and a 24 bp deletion in line 23-15. These mutations caused frameshifts leading to premature stop codons and truncated Lyc e1.01 proteins. In lines 23-15, the amino acid substitution and truncation occurred earlier in the sequence than in lines 23-9. (**D**) Predicted amino acid alterations resulting from 4 bp deletions in Lyc e1.02. The deletions induced a frameshift mutation that altered the downstream coding sequence, resulting in an extended protein length from 131 to 139 amino acids. Conserved, semi-conserved, and non-conserved residues are indicated by black, gray, and white backgrounds, respectively. Red color sequences indicate amino acid regions that differ between the mutant and WT proteins.

T_1_ line 23-9 harbored a deletion of 8 bp in *Lyc e1.01* and one of 4 bp in *Lyc e1.02*. T_1_ line 23-15 carried a deletion of 24 bp in *Lyc e1.01* and the same deletion of 4 bp in *Lyc e1.02* (Figure 3A,B). Because of these deletions, the predicted amino acid sequence of Lyc e1.01 in both lines was truncated compared to that in the wild type, whereas Lyc e1.02 was unexpectedly extended, resulting in a longer protein product (Figure 3C,D). The predicted protein sequences contained premature stop codons or truncations, which likely abolished profilin function. In these two mutants, the protein structures of Lyc e1.01 and Lyc e1.02 exhibited distinct alterations. In mutant 23-9, the number of α-helices at the C-terminal region was reduced compared to the control. In contrast, in mutant 23-15, the α-helices were replaced by β-sheets (Figure S3).

**Figure 4:**
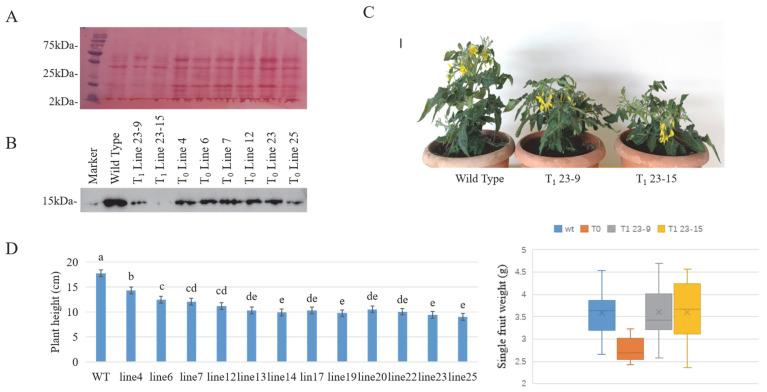
Profilin protein detection and phenotypic characterization. (**A**) Ponceau S staining of total protein extracted from tomato fruit and transferred to PVDF membranes, used as a loading control prior to immunodetection. (**B**) Immunoblot analysis using *Arabidopsis* anti-profilin-2 antibody: T_0_ samples were loaded at 40 μg per lane, and T_1_ and wild-type samples at 20 μg per lane. Immunoblot results showed a reduction in profilin protein levels in the mutant lines compared to the wild type. Notably, profilin protein was nearly undetectable in mutant T_1_ line 23-15. (**C**) Growth habit of 6-week-old tomato plants grown in a controlled plant growth room (25 °C, 16 h light/8 h dark photoperiod). Compared to wild-type plants, the profilin mutants exhibited reduced plant height. (Scale bar = 1 cm) (**D**) Comparison of tomato plant height and fresh fruit weight between wild-type and mutant lines grown in the greenhouse. Profilin mutants exhibited significantly reduced plant height compared with the wild type. Different lowercase letters indicate statistically significant differences among groups (n = 10 plants per group; *p* < 0.05). The fruit weight of T_1_ mutants showed no significant difference compared to the wild type. However, the fruit weight of T_0_ generation plants was significantly lower, likely due to weaker growth of callus-derived regenerants compared to seed-derived wild-type plants. (*p* < 0.05).

Profilin protein production was evaluated after protein extraction from the edited plants. Western blot analysis of T_0_ fruits showed residual profilin expression, likely due to mosaicism or incomplete editing (Figure 4A and Figure S5). In contrast, only a trace amount of profilin was detected in T_1_ line 23-15, indicating successful knockout. Line 23-9 still showed weak profilin bands, suggesting partial function retention due to the nature of the mutation. This difference may be attributed to the structural deletion of an α-helix in mutant 23-9 and the incorporation of a novel β-sheet structure in mutant 23-15, which may have disrupted protein function in the latter.

CRISPR/Cas9-mediated knockout of the tomato profilin gene resulted in reduced plant height (Figure 4C). Under greenhouse conditions, T_1_ mutant plants exhibited a significant decrease in height, from 17 cm (wild-type plants) to an average of 11 cm (*p* < 0.05; Figure 4D). Despite this growth phenotype, the mutants remained fertile and produced normal fruits, indicating that the targeted disruption of *Lyc e1* did not affect reproductive development. The fresh fruit weight of T_1_ mutant tomatoes was not significantly different from that of the wild type. However, in the T_0_ generation, the fruit weight was significantly lower than that of the wild type (Figure 4D). This reduction may be attributed to the fact that T_0_ regenerated plants were derived from callus tissues, which generally exhibit weaker growth than seed-derived plants, resulting in smaller fruit development.

In CRISPR/Cas9-based genome editing systems, off-target modifications can occur at genomic loci with high sequence similarity to the intended target site. Therefore, to assess the specificity of the sgRNA, we amplified and sequenced the most likely off-target gene, *Solyc03g083210.3*, from all T_0_ lines (Figure S4). Sanger sequencing revealed no evidence of peak overlap at this site, and the sequence was identical to that of the control. No mutations were detected at this locus, indicating that the sgRNA exhibited high target specificity.

## 3. Discussion

In this study, we successfully employed the CRISPR/Cas9 system to disrupt two key tomato allergen-encoding genes, *Lyc e1.01* and *Lyc e1.02*. By designing a single sgRNA targeting a conserved region shared by both genes, we generated loss-of-function mutations and identified two homozygous Cas9-free T_1_ lines (Table S1). Western blot analysis confirmed that the profilin protein was significantly decreased in line 23-15 (Figure 4B), validating this approach as an effective strategy for reducing tomato allergenicity.

Although both target sequences were identical, a marked difference in editing efficiency was observed in the protoplast assay: 6.1% for *Lyc e1.01* and 42.5% for *Lyc e1.02* (Table 2). This discrepancy may be attributed to differences in chromatin structure, epigenetic modifications, or genomic context. Chromatin accessibility influences Cas9 binding and cleavage efficiency [27,28,29]. Given that *Lyc e1.01* and *Lyc e1.02* are located on different chromosomes, variations in local chromatin states likely contributed to differential editing outcomes.

We also observed differences in profilin expression among homozygous mutants. Although line 23-15 exhibited significantly decreased profilin protein levels, line 23-9 retained detectable levels of profilin (Figure 4B). Structural modeling suggests that the mutation in 23-15 caused deletion of the C-terminal α-helix and replacement with the β-sheet, which is essential for profilin functions, including poly-L-proline binding and actin regulation [30,31,32]. In contrast, the profilin structure in mutant 23-9 only exhibited a reduction in the number of α-helices, with no introduction of novel structural elements, which may have led to incomplete functional inactivation of the protein. Profilin protein is still detectable in T_0_ mutants, but at reduced levels compared to wild-type. Furthermore, the loading amount in the T_0_ sample is twice that of the wild-type. Because T_0_ genotypes are often heterozygous, there is no guarantee that every mutation will be effective, resulting in a small amount of normal profilin protein. Although line 25 contains the least amount of profilin protein, no homozygous Cas9-free lines were detected in the T_1_ generation. Further selfing to obtain T_2_ lines can yield homozygous and effective Cas9-free mutants, as seen in line 23-15.

Notably, T_1_ mutant plants showed reduced plant height compared to wild-type controls, indicating a potential role of profilin in vegetative growth (Figure 4D). This phenotype is consistent with previous findings in *Arabidopsis* and tomatoes, where the RNA interference-mediated suppression of profilin resulted in dwarfism and impaired fruit development [9,33]. However, unlike earlier transgenic approaches that broadly suppressed multiple profilin genes, our CRISPR/Cas9-based strategy specifically targeted the allergenic isoforms *Lyc e1.01* and *Lyc e1.02*, minimizing pleiotropic effects. Despite the reduction in plant height, the mutant lines remained fertile, and fruit developed normally (Figure 4C). Since the Micro-Tom used in this experiment is a dwarf tomato variety, a decrease in plant height will inevitably have an impact on yield. Perhaps in tomato varieties with higher plant heights, the effect of reduced yield caused by the decrease in plant height will be mitigated.

Notably, the off-target analysis revealed no mutations at the highest-risk prediction site (*Solyc03g083210.3*) (Figure S4), supporting the specificity of our sgRNA design. As we know, the specificity of the CRISPR/Cas9 system is very high, but it also has risks. Before it can be put into use in breeding and production, a complete genome sequencing is still required. This is critical for the regulatory approval and consumer acceptance of genetically edited crops, particularly for applications in food allergy mitigation.

In this study, we demonstrated that CRISPR/Cas9-targeted editing of *Lyc e1.01* and *Lyc e1.02* genes resulted in significantly decreased levels of profilin, a major allergen, from tomato fruit. By designing a single sgRNA targeting a conserved region shared by both *Lyc e1.01* and *Lyc e1.02*, we generated homozygous Cas9-free mutants exhibiting significantly decreased profilin expression. The edited lines maintained their reproductive ability, and although plant height was reduced, fruit development was unaffected. Thus, CRISPR/Cas9-mediated gene editing effectively reduced allergenic profilin proteins without altering fruit size. The impact of these mutations on the overall crop yield needs to be investigated under field conditions.

The results of this study provide the first proof-of-concept for the development of hypoallergenic tomato cultivars through precise genome editing. This study represents a significant step toward the development of hypoallergenic tomato cultivars and highlights the broader potential of genome editing for food safety enhancement. Our approach offers a promising platform for improving food safety and may be extended to other allergenic genes in tomatoes or related *Solanaceae* crops. Future studies should include comprehensive allergenicity testing using patient sera and long-term agronomic evaluations to assess the field performance and safety of the edited lines.

## 4. Materials and Methods

### 4.1. Plant Material

The tomato cv. ‘Micro-Tom’ was used as the model plant owing to its short life cycle and suitability for transformation [34]. Seeds were surface-sterilized in 70% (*v*/*v*) ethanol prepared from absolute ethanol and sterile distilled water for 1 min, then in sodium hypochlorite solution (1% *v*/*v*, Sigma-Aldrich, St. Louis, MO, USA) for 30 min, and rinsed five times with sterile distilled water. Sterilized seeds were inoculated onto half-strength Murashige and Skoog (½ MS) solid medium (Duchefa Biochemie, Haarlem, The Netherlands) supplemented with 3% (*w*/*v*) sucrose and 0.8% (*w*/*v*) agar, with the pH adjusted to 5.8. Seeds were grown under controlled environmental conditions with a 16 h light/8 h dark photoperiod under temperature and relative humidity of 25 °C and 70–80%, respectively, and a light intensity of 98–117 μmol·m^−2^·s^−1^ provided by cool-white fluorescent lamps (FHF32SSEX-D, Osram GmbH, Munich, Germany).

### 4.2. sgRNA Design and Synthesis

A conserved target region shared by *Lyc e1.01* and *Lyc e1.02* was identified using online CRISPR/Cas9 design tools CRISPRdirect (https://crispr.dbcls.jp/, accessed on 4 March 2023). The sgRNA template was generated by PCR amplification, incorporating a T7 promoter sequence, and subsequently used for in vitro transcription with T7 RNA polymerase (New England Biolabs, Ipswich, MA, USA). Following transcription, the sgRNA was treated with RNase-free DNase I (New England Biolabs, Ipswich, MA, USA) at 37 °C for 15 min to remove residual DNA templates. The RNA was purified using the RNeasy MinElute Cleanup Kit (Qiagen, Hilden, Germany). The concentration and integrity of purified sgRNA were assessed using a NanoDrop spectrophotometer (Thermo Fisher Scientific, Waltham, MA, USA) and confirmed by 2% agarose gel electrophoresis.

### 4.3. Protoplast Isolation and Transformation

Cotyledons from one-week-old tomato seedlings were cut into strips measuring ~2 mm, digested in enzyme solution containing 1.2% Viscozyme^®^ (Novonesis, Bagsværd, Denmark), 0.6% PectinEX^®^ (Novonesis, Bagsværd, Denmark), and 0.6% Celluclast^®^ (Novonesis, Bagsværd, Denmark) in 0.4 M D-Mannitol (Sigma-Aldrich, St. Louis, MO, USA) with 8 mM calcium chloride (Sigma-Aldrich, St. Louis, MO, USA) and 0.5 mM MES solution (Sigma-Aldrich, St. Louis, MO, USA), then incubated overnight at 25 °C in the dark [23]. Protoplasts were filtered through a 70-μm cell strainer (SPL Life Sciences, Pocheon, Republic of Korea), centrifuged, and resuspended in W5 solution (154 mM NaCl, 125 mM CaCl_2_, 5 mM KCl, 2 mM MES, and 5 mM glucose), then incubated on ice for 30 min. Cas9 protein was purchased from ToolGen (ToolGen, Seoul, Republic of Korea), and sgRNAs were synthesized in our laboratory by PCR annealing using the designed oligonucleotide primers. Ribonucleoprotein (RNP) complexes were then assembled by incubating Cas9 protein with sgRNA at a molar ratio of 1:3, and the resulting complexes were delivered into protoplasts via PEG–Ca^2+^–mediated transfection [35]. Transformation efficiency was monitored using a GFP-expressing plasmid to evaluate the mutation rate and sgRNA validation, according to the method described by Yu [14]. Green fluorescence was observed under a fluorescence microscope 24 h after successful transformation of tomato protoplasts with the GFP plasmid.

### 4.4. Vector Construction and Stable Transformation

We used the CRISPR/Cas9 vector pKI1.1R (Addgene plasmid #85808; http://n2t.net/addgene:85808, accessed on 10 March 2023; RRID: Addgene_85808), containing an *RPS5A*-driven Cas9 and an *AtU6.26*-driven sgRNA cassette [36]. The sgRNA was cloned into the AarI site. Recombinant vectors were verified by Sanger sequencing and transformed into *Agrobacterium tumefaciens* GV3101 (C58C1 Rif^R^, pMP90RK) competent cells. Tomato cotyledon explants were infected with *Agrobacterium tumefaciens* suspension at an optical density (OD600) of 0.5 and cultured on MS medium supplemented with 3 mg/L 6-benzylaminopurine (Duchefa Biochemie, Haarlem, The Netherlands) and 0.2 mg/L indole-3-acetic acid (Duchefa Biochemie, Haarlem, The Netherlands). Hygromycin B (10 mg/L; Duchefa Biochemistry) was added to select transformed calli, and cefotaxime sodium (500 mg/L; MB Cell, Seoul, Republic of Korea) was used to suppress the growth of residual *Agrobacterium* after co-cultivation. The regenerated shoots were subsequently rooted and transferred to soil according to the method described by Chetty [37].

### 4.5. Genotyping and Western Blotting

Genomic DNA was extracted from young tomato leaves using the standard CTAB method [38]. PCR was performed to confirm the presence of the Cas9 transgene and amplify the target genomic regions. The PCR products were subjected to Sanger sequencing, and the resulting chromatograms were analyzed for mutation profiles using the ICE CRISPR Analysis Tool (https://ice.editco.bio/#/, accessed on 20 June 2024).

Total protein was extracted from freeze-dried tomato fruit tissues using NP-40 Lysis Buffer (250 mL; Thermo Fisher Scientific, Waltham, MA, USA). Western blotting was performed using an anti-profilin 2 primary antibody (1:2000 dilution; *Arabidopsis thaliana* PRF2, AT4G29350; Cat#PHY2385A; PhytoAB, San Jose, CA, USA) and goat anti-rabbit IgG H&L, HRP-conjugated (1:10,000 dilution; Cat# PHY6000; PhytoAB, San Jose, CA, USA). This primary antibody binding site is a conserved region of the KLH-conjugated synthetic peptide (19 aa from the central section) derived from *Arabidopsis thaliana* PRF2. Preliminary experiments showed that there was a clear band at 15 kDa, which is suitable for Western blotting experiments of tomato profilin. Equal protein loading was verified by Ponceau S staining (Thermo Fisher Scientific, Waltham, MA, USA) prior to immunodetection.

### 4.6. Off-Target Analysis

To assess the specificity of the CRISPR/Cas9 system and evaluate potential off-target effects, bioinformatics prediction of off-target sites was conducted using CRISPR-P 2.0 (http://crispr.hzau.edu.cn/CRISPR2/, accessed on 8 July 2024). Among the predicted sites, the candidate with the highest homology, located within the gene *Solyc03g083210.3*, was selected for experimental validation because of its susceptibility to off-target cleavage.

Genomic DNA was extracted from young leaves of all T_0_ plants. The genomic region corresponding to the predicted off-target site was amplified by PCR using site-specific primers designed to flank the candidate site with at least 200 bp on either side. The PCR products were purified and sequenced by Sanger sequencing. The resulting chromatograms were aligned to the wild-type reference sequence using BioEdit software version 7.2 (https://bioedit.software.informer.com, accessed on 8 July 2024) to identify sequence variations indicative of off-target editing.

### 4.7. Statistical Analysis

All experiments were performed with at least three independent biological replicates unless otherwise stated. Quantitative data, including sgRNA editing efficiency, transformation rates, protein expression levels, and plant height, are presented as the mean ± standard deviation (SD). Editing efficiency was determined based on the proportion of mutated sequences relative to total reads, as analyzed from Sanger sequencing chromatograms and deep sequencing data using the TIDE and ICE tools. Western blot band intensities were quantified using ImageJ software (version 1.53), with relative protein abundance normalized against internal loading controls (e.g., Ponceau S staining).

Statistical significance was evaluated using one-way analysis of variance (ANOVA) followed by Tukey’s post hoc test for multiple comparisons, or Student’s *t*-test for pairwise comparisons. A *p*-value of <0.05 was considered statistically significant.

## Figures and Tables

**Table 1:** Summary of sgRNA target sites designed for *Lyc e1* genes.

Gene	Position	Strand	Target Sequence	Sequence Information		Out-of-Frame Score	Mismatch 0	Mismatch 1	Mismatch 2
	Start-End	(+/−)	(20mer + PAM)	GC% (%)	TM (°C)				
*Lyc e1*	346–368	+	AATATGATTGTTGAAAGGTTGGG	25.00%	59.46	62.6	2	1	0

**Table 2:** Editing efficiencies and indel mutation types of *Lyc e1.01* and *Lyc e1.02* genes detected in tomato protoplasts.

Gene	Mutation in the Target Site	Indel	Indel (%)	Mutated Reads	Total Sequence Reads
	AATATGATTGTTGAAAGGTTGGG *				
*Lyc e 1.01*	AATATGATTGTTGAAAGGGTTGGG	+1	2.31%	204	
	AATATGATTGTTG—AAGGTTGGG	−1	2.20%	194	
	AATATGATTGTTGAAAAGGTTGGG	+1	0.68%	60	
			6.10%	536	8836
*Lyc e 1.02*	AATATGATTGTTGAAA—GTTGGG	−1	12.92%	3044	
	AATATGATTGTTGAAAG—TTGGG	−1	5.75%	1356	
	AATATGATTGTT———GGTTGGG	−4	1.93%	456	
			42.50%	10,019	23,569

* A conserved sequence of *Lyc e1.01* and *Lyc e1.02* genes targeted by the designed sgRNA in the wild-type tomato. Note: Red letters indicate nucleotide positions where base changes (insertions or deletions) occurred in the mutant.

**Table 3:** Actual mutation frequency and type in T_0_ transgenic lines regenerated from tomato cotyledon culture.

Target Gene	Total Examined Explants	Regenerated Shoots	Mutated Plants	Mutation Frequency (%)	Biallelic/Homozygous (%)	Monoallelic (%)	Chimeric (%)
*Lyc e1.01*	71	25	13	52	15.4	0	84.6
*Lyc e1.02*					7.7	0	92.3

**Table 4:** Editing efficiencies and allele changes in *Lyc e1.01* and *Lyc e1.02* of 13 T_0_ transgenic lines out of a total of 25 regenerated tomatoes.

Gene	Mutation Rate and Allele Types of the Regeneration T_0_ Lines												
	4	6	7	12	13	14	15	17	19	20	22	23	25
*Lyc e1.01*	96%	81%	61%	84%	88%	58%	80%	85%	77%	82%	84%	91%	89%
	−1, −2 *	−8, −24, −24	−1, −12, WT	−8, −24, −24	−8, −16, −23, −24	−1, −12, WT	−1, −3, −4, WT	−8, −24, −24	−9, −25, −9	−8, −24, −24	−8, −24, −24	−8, −24, −24	−2, −11
*Lyc e1.02*	92%	95%	52%	96%	83%	47%	47%	86%	90%	49%	89%	95%	87%
	−1, −8, WT	−1, −4, −6	−1, −4, WT	−1, −4, −5	−2, −5, WT	−2, −4, WT	+1, −1, WT	−1, −4, −11, WT	+1, −1, −3, −4, WT	−2, −4, −18, −25, WT	+1, −1, −2, −4	−1, −4	−1, −2, −5, −9, −18, WT

* Signs of minus (−) and plus (+) denote deletion and insertion of bases, respectively, with the numbers indicating nucleotides changed compared to wild-type alleles. The same numbers in the single line refer to different sequences with the same length of change.

## Data Availability

The data generated and/or analyzed during the current study are available from the corresponding author upon request.

## Supplementary Materials

The following supporting information can be downloaded at: https://www.mdpi.com/article/10.3390/plants14243837/s1. Figure S1: Fluorescence, bright-field, and merged images of GFP-expressing protoplasts by PEG-Ca+ transfer method. Evaluate the transformation efficiency based on the proportion of fluorescent cells, 105 out of 164 protoplasts (64.02%) showed GFP fluorescence. Scale bar = 100 μm; Figure S2: PCR amplification of the Cas9 transgene in the T_1_ generation. Out of 240 individuals, 50 lacked detectable Cas9 amplification (20.8%), indicating transgene-free status. The observed segregation ratio was inconsistent with Mendelian expectations, possibly due to multiple independent T-DNA insertions in the T_0_ generation; Figure S3: Predicted secondary structures of mutant profilin proteins (Lyc e1.01) in lines 23-9 and 23-15, respectively, generated using the SWISS-MODEL server (https://swissmodel.expasy.org/interactive). In mutant line 23-9, the number of α-helices at the C-terminal region was reduced compared to the wild-type structure. In mutant line 23-15, α-helical structures were replaced by β-sheets, indicating a more substantial conformational change that may lead to protein inactivation; Figure S4: Sanger sequencing results for the predicted off-target site in the *Solyc03g083210.3* gene. No sequence variations were detected when compared to the wild-type control, indicating high specificity of the CRISPR/Cas9 system; Figure S5: Preliminary test of the anti-profilin 2 antibody in tomato fruits. Total protein was extracted from freeze-dried tomato fruit powder. The amount loaded per lane was based on the dry weight of the fruit sample; Table S1: The genotypes of editing sites in T_1_ T-DNA Free mutants.
