# Supplementary material for: CRISPR/Cas9-Targeted Gene Editing of Allergenic Profilin-Encoding Lyc e1 in Tomato Fruit
Source: Plants (Basel). 2025 Dec 16;14(24):3837. doi: 10.3390/plants14243837 (PMC12736926; doi:10.3390/plants14243837)
Supplement: Supplementary file 1 [file plants-14-03837-s001.zip › plants-4007489-supplementary.pdf]

Supplementary Table S1. The genotypes of editing sites in T_1 T-DNA Free mutants.

No. T_1	*Lyc e1.01*	*Lyc e1.02*	No. T_1	*Lyc e1.01*	*Lyc e1.02*
	Genotype	Genotype		Genotype	Genotype
4-3	-2	-1 | WT	17-19	-8 | -24	-1 | -2
4-14	-1 | -2	-1 | WT	19-4	-9 | -25	-1 | -4
4-18	-1 | -2	-1 | -8	19-5	-9 | -25	-1 | -4
6-13	-8 | -24	-1 | -4	19-12	-9 | -25	-1 | -4
7-10	-1 | -12	-4 | WT	19-13	-24	-2 | -4
7-13	-1 | -12	-2 | WT	19-15	-8 | -24	-2 | -4
7-18	-1	-2 | -4	19-19	-8	-2 | -4
7-19	-12	-2 | -4	19-20	-24	-2 | -4
12-2	-8 | -24	-1 | -5	20-6	-8 | -24	-2 | -4
12-9	-24	-1 | -5	20-7	-8	-2 | -4
12-14	-8 | -24	-1 | -5	20-9	-24	-2 | -4
12-15	-8 | -24	-1 | -5	20-10	-8	-2 | -4
13-2	-8 | -24	-2 | -5	20-15	-8 | -24	-2 | -4
13-4	-8	-2 | WT	22-4	-8 | -24	-2 | -4
13-9	-8 | -24	-2 | -5	22-10	-24 | -24	-1 | -2
13-12	-8	-5 | WT	22-12	-8 | -24	-1 | -4
13-15	-24	-2 | -5	23-2	-8 | -24	-1 | -4
14-1	-1	-4 | WT	23-3	-8 | -24	-1 | -4
14-8	-1 | -12	-2 | WT	23-6	-8 | -24	-4
14-9	-1	-2 | -4	23-9	-8	-4
14-16	-1	-2 | WT	23-10	-8 | -24	-4
14-18	-1	-2 | -4	23-15	-24	-4
17-1	-8	-1 | -4	23-19	-8 | -24	-4
17-2	-8	-1 | -4	25-3	-2 | -11	-18 | WT
17-5	-8	-1 | -2	25-6	-11	-9 | -18

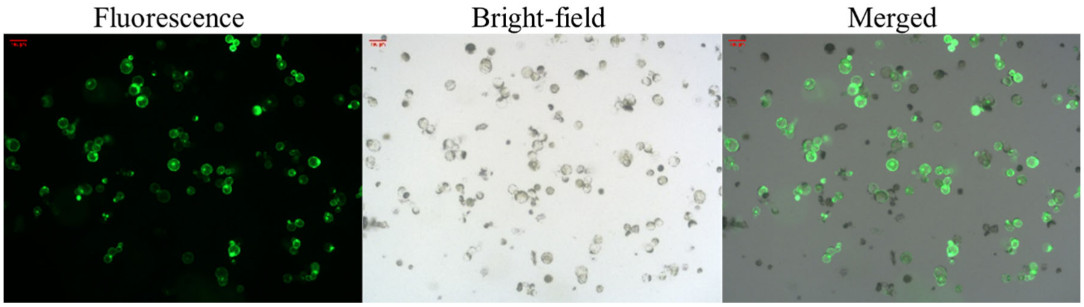

Supplementary Figure S1. Fluorescence, bright-field, and merged images of GFP-expressing protoplasts by PEG-Ca^{+} transfer method. Evaluate the transformation efficiency based on the proportion of fluorescent cells, 105 out of 164 protoplasts (64.02%) showed GFP fluorescence. Scale bar = 100 μm.

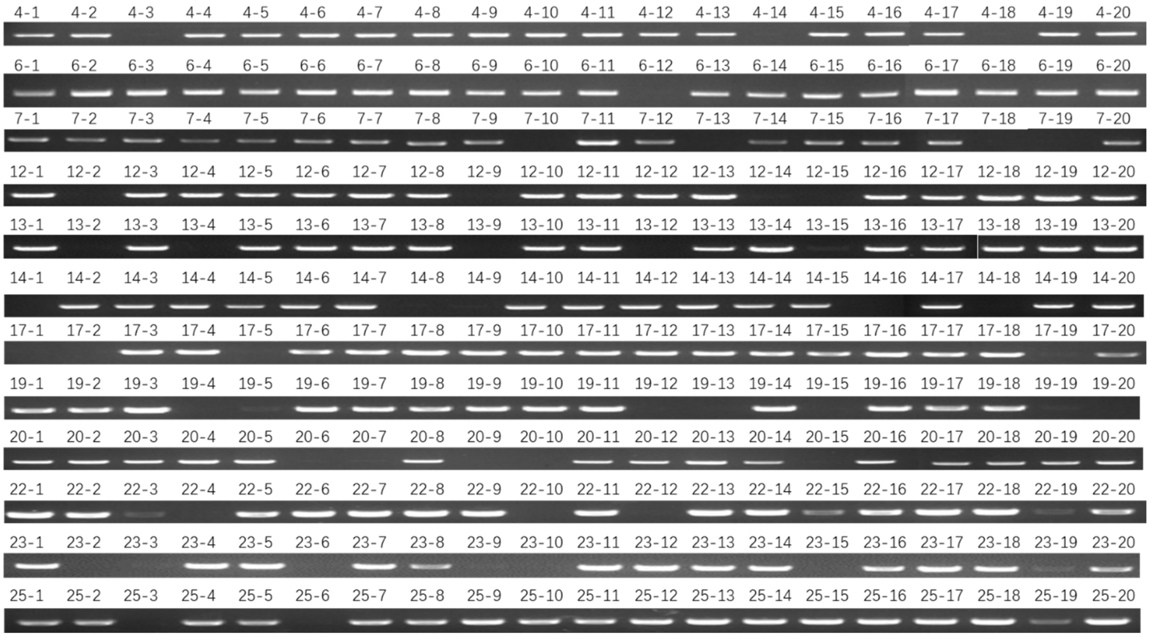

Supplementary Figure S2. PCR amplification of the Cas9 transgene in the T_1 generation.
Out of 240 individuals, 50 lacked detectable Cas9 amplification (20.8%), indicating transgene-free status. The observed segregation ratio was inconsistent with Mendelian expectations, possibly due to multiple independent T-DNA insertions in the T_0 generation.

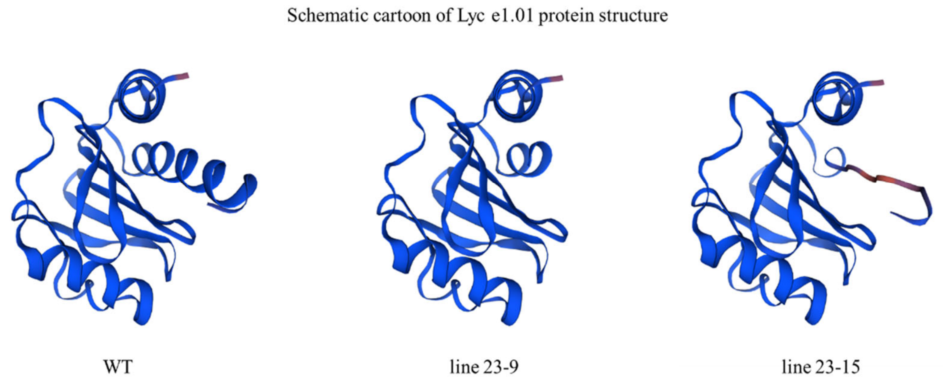

Supplementary Figure S3. Predicted secondary structures of mutant profilin proteins (*Lyc e1.01*) in lines 23-9 and 23-15, respectively, generated using the SWISS-MODEL server (https://swissmodel.expasy.org/interactive). In mutant line 23-9, the number of α-helices at the C-terminal region was reduced compared to the wild-type structure. In mutant line 23-15, α-helical structures were replaced by β-sheets, indicating a more substantial conformational change that may lead to protein inactivation.

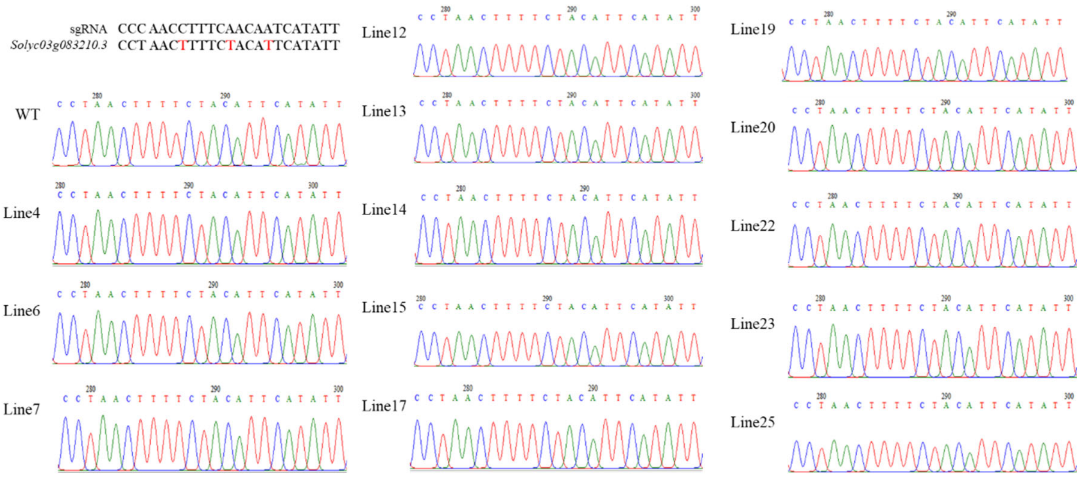

Supplementary Figure S4. Sanger sequencing results for the predicted off-target site in the *Solyc03g083210.3* gene. No sequence variations were detected when compared to the wild-type control, indicating high specificity of the CRISPR/Cas9 system.

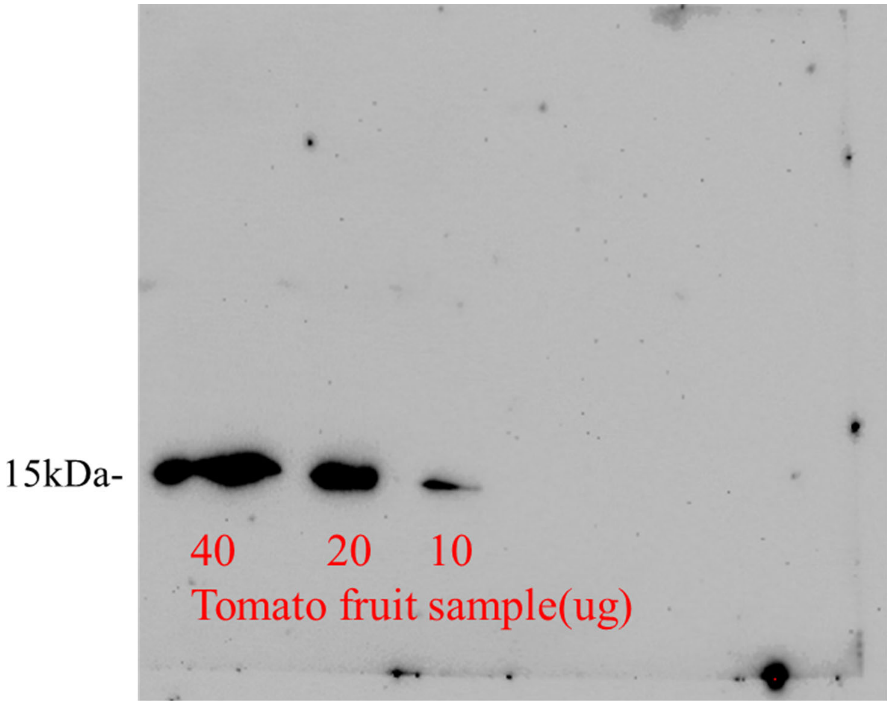

Supplementary Figure S5. The preliminary experiment of anti-profilin 2 antibody in tomato fruits. Total protein was extracted from freeze-dried tomato fruit powder. The amount load-ed per lane was based on the dry weight of the fruit sample.
